# Reduced Platelet MAO-B Activity Is Associated with Psychotic, Positive, and Depressive Symptoms in PTSD

**DOI:** 10.3390/biom12050736

**Published:** 2022-05-23

**Authors:** Senka Repovecki, Gordana Nedic Erjavec, Suzana Uzun, Lucija Tudor, Matea Nikolac Perkovic, Marcela Konjevod, Oliver Kozumplik, Dubravka Svob Strac, Zrnka Kovacic Petrovic, Ninoslav Mimica, Nela Pivac

**Affiliations:** 1 University Psychiatric Hospital Vrapce, 10000 Zagreb, Croatia; srepovecki@gmail.com (S.R.); suzana.uzun@gmail.com (S.U.); okozumplik@hotmail.com (O.K.); zrnka.kovacic@gmail.com (Z.K.P.); nino.mimica@gmail.com (N.M.); 2 Laboratory for Molecular Neuropsychiatry, Division of Molecular Medicine, Ruder Boskovic Institute, 10000 Zagreb, Croatia; gnedic@irb.hr (G.N.E.); ltudor@irb.hr (L.T.); mnikolac@irb.hr (M.N.P.); mkonjev@irb.hr (M.K.); dsvob@irb.hr (D.S.S.); 3 School of Medicine, University of Zagreb, 10000 Zagreb, Croatia

**Keywords:** PTSD, platelet MAO-B activity, clinical symptoms, PANSS, CAPS

## Abstract

Post-traumatic stress disorder (PTSD) is a trauma-related disorder. Platelet monoamine oxidase (MAO-B) is a peripheral biomarker associated with various symptoms in different psychopathologies, but its role in PTSD or different symptoms in PTSD is not clear. This study elucidated the association between platelet MAO-B activity and clinical symptoms occurring in PTSD. Platelet MAO-B activity was determined in 1053 male Caucasian subjects: 559 war veterans with PTSD (DSM-5 criteria), 62 combat exposed veterans who did not develop PTSD, and 432 non-combat exposed healthy controls. Clinical symptoms in PTSD were determined using CAPS and PANSS. Platelet MAO-B activity, controlled for the effect of smoking, was significantly increased in PTSD with severe versus mild and moderate traumatic symptoms, and was significantly decreased in PTSD subjects with severe versus mild positive, psychotic, and depressive symptoms. This finding was further confirmed with reduced platelet MAO-B activity in PTSD veterans with severe versus mild individual items of the PANSS-depressed, PANSS-psychotic, and PANSS-positive subscales. Altered platelet MAO-B activity, controlled for the possible confounders, was associated with the development and severity of different symptoms occurring in PTSD. These findings confirmed the role of platelet MAO-B activity as a peripheral marker of various psychopathological symptoms.

## 1. Introduction

Post-traumatic stress disorder (PTSD) is a severe trauma- and stress-related disorder [1] that develops in only a subset of vulnerable individuals [2] exposed directly or indirectly to a traumatic event. Trauma includes either a single traumatic event or a prolonged exposure to a stressful event or events [1]. PTSD might also develop when a person witnesses a traumatic event that happens to their family members or close friends, or in professional workers exposed to the consequences of traumatic events [1]. As not all individuals who have experienced extreme traumas will develop PTSD, it is important to determine its neurobiological underpinning. Namely, numerous neurobiological, genetic, glycomic, metabolomic, epigenetic, psychological, cognitive, emotional, environmental, and psychosocial factors, as well as complex interactions between them, seem to determine resilience or vulnerability to PTSD [3,4,5,6,7,8,9,10]. Risk factors, manifested through diverse biomarkers [11], might predict the development of PTSD [12] or the development of more severe symptoms in PTSD [13]. Numerous biomarkers are listed and organized in the meta-data in the PTSD Biomarker Database [11], associated with the neurotransmitters dopamine, noradrenaline, and serotonin [14]; neurotrophic factors such as brain derived neurotrophic factor (BDNF) [15]; the hypothalamic-pituitary-adrenal (HPA) axis; and the immune system [16,17]. Owing to the complexity of PTSD and its symptoms, at present, there are no specific and validated biomarkers for PTSD [12] or for diverse symptoms occurring in PTSD.

The importance of the dopamine system in PTSD is confirmed through its role in mood, emotions, behavior, attention, vigilance, emotional abnormalities, arousal and sleep, and reward learning deficits, which are all dysregulated in PTSD. Trauma is related to a reduction in dopaminergic neural activity in humans and in animal models [18]. Not only catechol-o-methyl transferase (COMT), but also the enzymes monoamine oxidase type A (MAO-A) and type B (MAO-B), degrade dopamine [19,20]. MAO-B is widely distributed in most human tissues including the central nervous system (CNS) and platelets. In humans, platelet MAO-B shares the same amino acid sequence with MAO-B in the brain [21]. Additionally, platelets contain some components of the serotonergic system and were suggested to present a peripheral model to study serotonergic abnormalities in psychiatric disorders [22,23,24,25,26]. Platelets are also described as substantial indicators of neurologic diseases as they enter the compromised blood brain barrier; infiltrate CNS; and release serotonin, BDNF, and other neuroinflammatory and oxidative stress factors [27].

Both human [28] and animal [29] studies confirmed the important role of MAO in aggressive behavior, assumed to be achieved by modulating corticolimbic circuits affecting social information processing and emotional responsiveness. Platelet MAO-B activity was reported to be reduced in different psychopathological and disinhibited, criminal, and violent behaviors and aggression [21,30,31,32,33]. However, opposing data also exist [34,35]. Increased platelet MAO-B activity was found in aggressive male youth living in juvenile detention, which was not associated with delinquent behavior [34], or in adult male subjects with chronic alcohol dependence, which was not affected by alcohol-related phenotypes [35]. There are also conflicting findings on the association between MAO-B and PTSD [36,37]. Reduced platelet MAO-B activity in PTSD versus controls [36], or similar platelet MAO-B activity in war veterans with or without PTSD [37], was reported. Increased platelet MAO-B activity was found in veterans with psychotic versus non-psychotic PTSD, in both smokers and non-smokers [38]. This finding was later confirmed in larger groups of veterans with PTSD, but only in smokers, not in non-smokers [39]. A significant correlation was detected between the Clinician-Administered PTSD Scale (CAPS) scores and platelet MAO-B activity [39]. Agitation, which is frequent in veterans with PTSD [40], determined by the Positive and Negative Syndrome Scale (PANSS) excitement subscale scores, was associated with either reduced (in smokers) or unaltered (in non-smokers) platelet MAO-B activity [39]. These divergent findings might be explained by the several factors that may influence MAO-B enzyme activity [31], but primarily by the small sample sizes in the previous studies.

As MAO-B degrades dopamine, alterations in MAO-B activity can lead to higher/lower dopamine availability in the prefrontal cortex, which might be associated with more or less pronounced symptoms developed during the time-course of PTSD. The present study aimed to elucidate the association of platelet MAO-B activity with the severity of different symptoms occurring in PTSD. The study included a large group (*N* = 559) of Croatian male war veterans with PTSD exposed to a similar combat experience. As platelet MAO-B activity is affected by smoking [39], alcohol dependence [35], sex [35], and various medication [31], as well as by liver diseases (cirrhosis, fibrosis, and sclerosis, and hepatocellular carcinoma) [35], in this study, we controlled platelet MAO-B activity, in the large number of subjects (*N* = 1053), for these possible confounders that were reported to influence platelet MAO-B activity [31,35,38,39,41].

The hypothesis of the study was that platelet MAO-B activity is significantly associated with severe traumatic, psychotic, excited, and positive symptoms in veterans with PTSD.

## 2. Materials and Methods

### 2.1. Participants

The study included 1053 unrelated male Caucasian individuals of Croatian origin, subdivided into three groups: 559 war veterans with combat-related PTSD, 62 combat exposed veterans who did not develop PTSD, and 432 non-combat exposed healthy male control subjects. All of the subjects were sampled between 2015 and 2017 in the University Psychiatric Hospital Vrapce, Zagreb, Croatia. War veterans were soldiers in the Homeland war in Croatia (1991–1995) and were exposed to multiple combat-related traumatic events. War veterans without PTSD and healthy control subjects were evaluated with the same diagnostic instruments and followed the same inclusion/exclusion criteria as subjects with PTSD. Prior to their involvement in the study, all subjects gave their written informed consent and compliance to fulfill psychiatric questionnaires. Moreover, all of the included subjects had to be medication-free for at least 30 days, except for low doses of benzodiazepines as needed. PTSD was diagnosed according to the Structured Clinical Interview (SCID) based on DSM-5 criteria [1]. The International Classification of Diseases (ICD-10) criteria was used in all participants to exclude potential liver-related disease such as fibrosis, sclerosis, cirrhosis, and malignant liver disease (K70.3; K70.2; C22.0). Additional exclusion criteria were major depression, schizophrenia, bipolar disorder, adult ADHD, Alzheimer’s disease, chronic drug abuse, alcohol dependence, current or recent (previous month) use of MAO-B inhibitors and other medication affecting platelet MAO-B activity, and the diagnosis of PTSD in healthy control subjects. The aims and procedures were approved by the Ethics Committee of the University Psychiatric Hospital Vrapce, Zagreb, Croatia and were explained in detail to all participants, who gave written informed consent. All procedures contributing to this work comply with the ethical standards of the relevant national and institutional committees on human experimentation and with the Helsinki Declaration of 1975, as revised in 2008.

The severity of PTSD was assessed using the Clinician-Administered PTSD Scale (CAPS) [42]. Scores for individual CAPS items in the group of war veterans with PTSD are given in Table 1.

Other clinical symptoms occurring in PTSD were assessed using the Positive and Negative Syndrome Scale (PANSS) [43], consisting of 30 items scored from 1 to 7 (1 = absent; 2 = minimal; 3 = mild; 4 = moderate; 5 = moderate severe; 6 = severe; 7 = extreme symptoms). Symptoms that scored ≥ 4 were considered as severe symptoms in the PANSS positive subscale (P1 + P2 + P3 + P4 + P5 + P6 + P7; cutoff = 28); the PANSS-psychotic subscale: including items P1 + P2 + P3 + P6, cutoff = 16 [39]; and the PANSS-depressed subscale: including items G1 + G2 + G3 + G6, cutoff = 16 [44]. For the PANSS-excitement subscale including items: P4 + P7 + G4 + G8 + G14, cutoff was set to 14 [45]. For the PANSS total scores as well as PANSS negative symptoms, no veterans with PTSD had a score lower than 4. Veterans with PTSD were evaluated using PANSS subscales as listed in Table 2.

As smoking (≥10 cigarettes per day) significantly decreases platelet MAO-B activity [38,39,41], all subjects were subdivided into smokers (*N* = 544) and non-smokers (*N* = 509).

### 2.2. Blood Sampling

Blood sampling was performed during the regular check-ups in the morning after overnight fasting, around 8 a.m. Blood samples (8 mL) were drawn in a plastic syringe with 2 mL of acid citrate dextrose anticoagulant. Platelet-rich plasma (PRP) was obtained after centrifugation of whole blood at 1810× *g* for 3 min and platelets were precipitated by further centrifugation of PRP at 5030× *g* for 15 min in a refrigerated centrifuge. The platelet pellet was washed with saline and centrifuged again. The obtained platelets were stored at −20 °C.

### 2.3. Determination of Platelet MAO-B Activity

Platelet MAO-B activity was determined with a spectrophotoflurimetric method (as described previously in [39] as a modification of Krajl’s [46] method). Briefly, standard (4-hydroxyquinoline, 4-HOQ), blank (water), and platelet sonicates were incubated with MAO-B substrate kynuramine at 37 °C. After 1 h, the reaction was stopped by adding cold 1 M NaOH. Spectrophotofluorimeter Varian Cary Eclipse was used for the measurement of 4-HOQ fluorescence, a product of kynuramine (exciting λ = 310 nm, emitted λ = 380 nm). The protein level was determined in platelets by the method of Lowry et al. [47]. Platelet MAO-B activity was expressed in nmol 4-OHQ/mg protein/h.

### 2.4. Statistical Analysis

The results were evaluated with SigmaStat 3.5 (Jandel Scientific Corp., San Jose, CA, USA). The normality of the distribution was assessed with the Kolmogorov–Smirnov test. The results are expressed as numbers and percentages, as well as median and 25th (Q1) and 75th (Q3) percentiles, and evaluated using the Mann–Whitney U test for two groups and Kruskal–Wallis ANOVA of ranks for three or more groups with Dunn’s post hoc test and Spearman’s coefficient of correlation. Multiple regression analysis was performed with platelet MAO-B activity as the dependent variable, and age and diagnosis as independent variables. All tests were two-tailed. To avoid correction for multiple testing and the issues related to multiple testing [48], all statistical tests were applied to cutoffs that were determined in advance: clinical symptoms in PTSD were evaluated using the CAPS and PANSS subscale cutoffs, as described above.

All tests were two-tailed and α was set at 0.05. G*Power 3 Software [49] was used to conduct power analyses, i.e., to determine a priori sample size and actual power. Statistical power was set to 0.800. For multiple regression (with expected small effect size = 0.02, two predictors), the required sample size was 485. For the Mann–Whitney U test (with expected small effect size (ω = 0.3), the required sample size was 278. For the Kruskal–Wallis ANOVA (with α = 0.05, a small effect size (ω = 0.20), and three groups), the total desired sample size was 246. As the study included 1053 individuals, it has an adequate sample size to determine significant differences in platelet MAO-B activity.

## 3. Results

Multiple regression analysis (F = 7.33; *p* = 0.001; Radj2 = 0.012) revealed no effect of age (β = 0.018; *p* = 0.551) and a significant effect of diagnosis (β = −0.117; *p* < 0.001) on platelet MAO-B activity.

**Figure 1:**
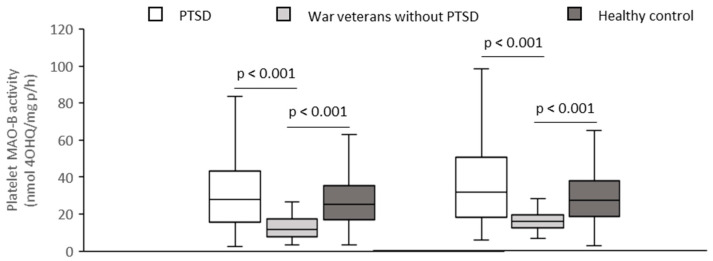
Platelet MAO-B activity in subjects with and without PTSD and healthy control subjects, subdivided according to smoking status.

Platelet MAO-B activity was significantly (Kruskal–Wallis ANOVA) increased (*p* < 0.001, Dunn’s test) in smokers (H = 24.60; *p* < 0.001) and in non-smokers (H = 39.53; *p* < 0.001) with PTSD when compared with corresponding veterans without PTSD, but did not differ when compared with control subjects (Figure 1).

**Figure 2:**
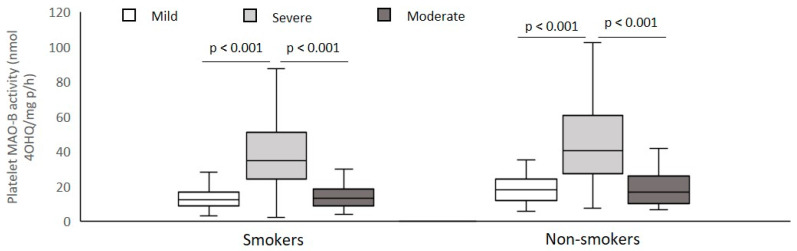
Platelet MAO-B activity in subjects with mild, moderate, and severe PTSD symptoms subdivided according to smoking status.

Veterans with PTSD were subdivided into smokers and non-smokers with mild, moderate, and severe PTSD symptoms, according to the CAPS scores. There were 43 veterans (7.7%) with PTSD with mild symptoms (with 46–65 scores), 121 veterans (21.6%) with PTSD with moderate symptoms (with 66–95 scores), and 395 veterans (70.7%) with PTSD with severe (with 96–136 scores) traumatic symptoms. Platelet MAO-B activity was significantly (Kruskal–Wallis ANOVA) increased (*p* < 0.001, Dunn’s test) in smokers (H = 134.96; *p* < 0.001) and in non-smokers (H = 60.67; *p* < 0.001) with severe symptoms compared with veterans with mild or moderate traumatic symptoms (Figure 2).

Spearman correlation revealed significant positive correlations between platelet MAO-B activity and total scores according to CAPS criteria B (σ = 0.50, *p* < 0.001), C (σ = 0.53, *p* < 0.001), and D (σ = 0.58, *p* < 0.001) in smokers. Moreover, in non-smokers, significant correlations between platelet MAO-B activity and total scores according to CAPS criteria B (σ = 0.44, *p* < 0.000), C (σ = 0.54, *p* < 0.000), and D (σ = 0.54, *p* < 0.000) were observed.

**Figure 3:**
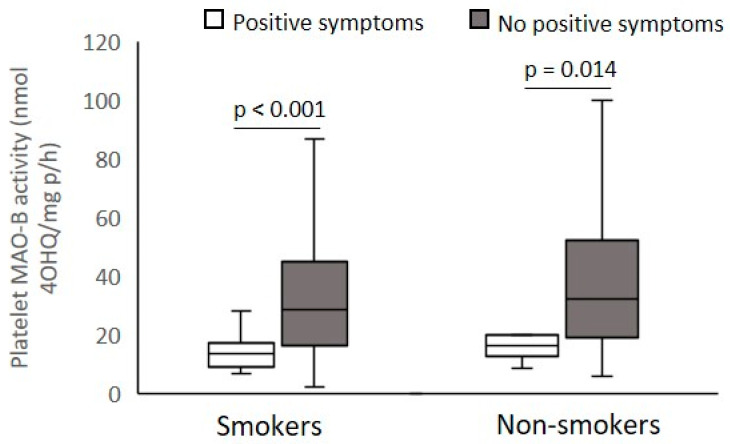
Platelet MAO-B activity in subjects with severe and mild symptoms evaluated by the PANSS-positive subscale, subdivided according to smoking status.

**Figure 4:**
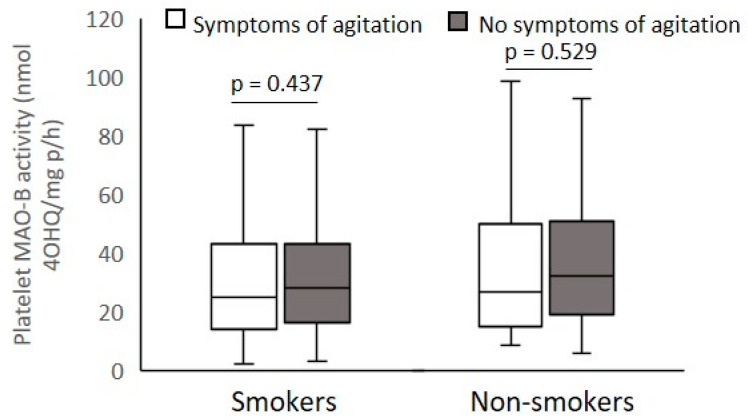
Platelet MAO-B activity in subjects with severe and mild symptoms evaluated by the PANSS-excitement subscale, subdivided according to smoking status.

**Figure 5:**
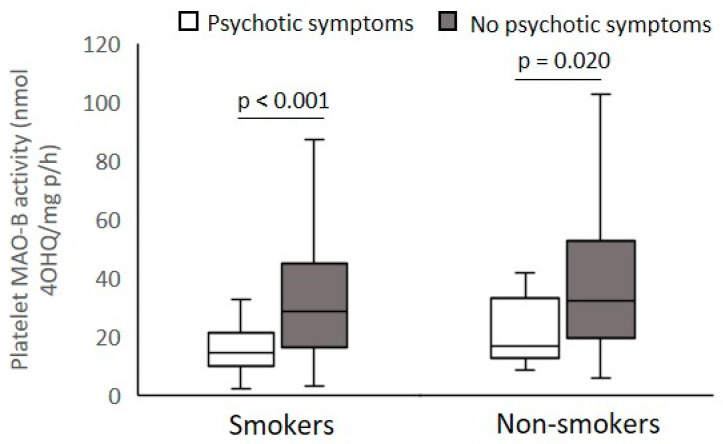
Platelet MAO-B activity in subjects with severe and mild symptoms evaluated by the PANSS-psychotic subscale, subdivided according to smoking status.

**Figure 6:**
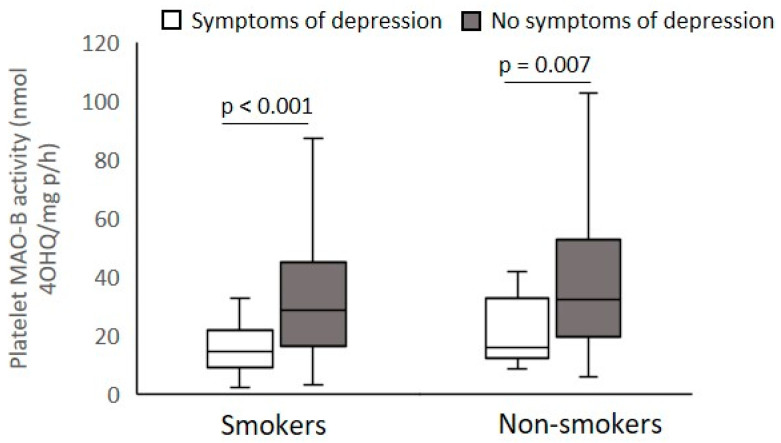
Platelet MAO-B activity in subjects with severe and mild symptoms evaluated by the PANSS-depressed subscale, subdivided according to smoking status.

According to the PANSS scores presented in Table 2, veterans with PTSD were subdivided into smokers and non-smokers with mild or severe PANSS positive symptoms (Figure 3), PANSS excitement/agitation symptoms (Figure 4), PANSS psychotic symptoms (Figure 5), and PANSS depression symptoms (Figure 6), respectively.

Platelet MAO-B activity was significantly (Mann–Whitney test) decreased in smokers (U = 6072.0; *p* < 0.001) and non-smokers (U = 997.0; *p* < 0.014) with severe symptoms compared with veterans with mild PANSS positive symptoms (Figure 3). According to the PANSS-excitement subscale, there were no significant differences (Mann–Whitney test) in platelet MAO-B activity within smokers (U = 11849.9; *p* = 0.437) or non-smokers (U = 2782.0; *p* = 0.529) with severe and mild pronounced agitation symptoms (Figure 4). Significantly reduced platelet MAO-B activity (Mann–Whitney test) was detected in smokers (U = 7494.5; *p* < 0.001) and non-smokers (U = 17046.0; *p* < 0.020) with symptoms severe compared with veterans with mild PANSS-psychotic symptoms, according to the cutoffs in the PANSS-psychotic subscale (Figure 5). In addition, significantly (Mann–Whitney test) decreased platelet MAO-B activity was also found in veterans with PTSD with more pronounced symptoms, i.e., severe depressive symptoms, compared with platelet MAO-B activity in those with mild depressive symptoms, in both smokers (U = 8964.0; *p* < 0.001) and nonsmokers (U = 1876.0; *p* = 0.007) (Figure 6).

To confirm the results obtained by comparing platelet MAO-B activity between war veterans with PTSD with mild and severe symptoms evaluated by PANSS subscales, platelet MAO-B activity was additionally evaluated in veterans with severe symptoms compared with those with mild symptoms according to the individual items of each PANSS subscale in smokers (Table 3) and non-smokers (Table 4).

In smokers and non-smokers with PTSD, individuals with severe symptoms according to the P1 (delusions), P2 (conceptual disorganisation), P3 (hallucinatory behavior), and P4 (excitement) items had significantly lower platelet MAO-B activity than smokers and non-smokers with PTSD with corresponding mild symptoms. Therefore, reduced platelet MAO-B activity found in smokers and non-smokers with psychotic (items P1 + P2 + P3 + P6) and positive (items P1 + P2 + P3 + P4 + P5 + P6 + P7) symptoms was induced by the significantly lower platelet MAO-B activity associated with delusions, conceptual disorganisation, hallucinatory behavior, and excitement (Table 3 and Table 4). Platelet MAO-B activity did not differ significantly within smokers or non-smokers subdivided according to the severity of other PANSS-positive or -psychotic symptoms (P5—grandiosity, P6—suspiciousness/persecution, and P7—hostility).

Regarding the PANSS excitement subscale items (Table 3 and Table 4), out of P4 + P7 + G4 + G8 + G14 items, significantly reduced platelet MAO-B activity in smokers and non-smokers with severe compared with mild P4 (excitement) scores, but similar MAO-B activity within those with P7 (hostility) scores, was found. No individuals had scores less than 4 on the G8 (uncooperativeness) item. Platelet MAO-B activity was significantly reduced in smokers and non-smokers with severe G4 (tension) symptoms and in smokers with severe G14 (poor impulse control) symptoms compared with those with mild symptoms. In non-smokers, platelet MAO-B did not differ between those with severe or mild G4 (tension) symptoms. In accordance, severe excitement or agitation was not significantly associated with platelet MAO-B activity in smokers and non-smokers with PTSD.

Regarding the PANSS depressive subscale items (Table 3 and Table 4), platelet MAO-B activity was significantly lower in smokers and non-smokers with severe G2 (anxiety) and G6 (depression) symptoms when compared with those with mild symptoms, but was similar in those with severe or mild G1 (somatic concern) and G3 (guilty feelings) symptoms. These results suggest that decreased platelet MAO-B activity found in smokers and non-smokers with severe depressive symptoms was due to the significantly lower platelet MAO-B activity associated with symptoms of anxiety and depression.

## 4. Discussion

The major finding of this study was that platelet MAO-B activity was significantly reduced in veterans with PTSD who had developed severe positive, psychotic, and depressive symptoms compared with veterans with PTSD who had mild positive, psychotic, and depressive symptoms, determined using the PANSS subscale scores. On the other hand, platelet MAO-B activity was significantly increased in veterans with PTSD with severe traumatic symptoms compared with those with mild or moderate traumatic symptoms, determined using the CAPS. These results were confirmed in both smokers as well as non-smokers, and suggest that altered platelet MAO-B activity is associated with different clinical symptoms in PTSD.

This study was performed to confirm or reject previous findings of the altered platelet MAO-B activity in PTSD versus controls, and in individuals with PTSD with more pronounced psychotic, positive, or agitated symptoms in PTSD versus those without these symptoms. In contrast to previous studies, this study included a large number of subjects (*N* = 1053), and platelet MAO-B activity was controlled for possible confounders such as sex, smoking, liver diseases, ethnicity, medication, and presence of different symptoms [31,35,38,39,41,50,51]. Our previous study [35] that included large groups of subjects with alcohol use disorder (AUD) revealed that liver diseases (cirrhosis, fibrosis, and sclerosis, as well as hepatocellular carcinoma) significantly reduced platelet MAO-B activity; therefore, in the present, study liver diseases were used as exclusion criteria. Female sex is associated with higher platelet MAO-B activity [35], and to exclude this effect, the present study included only male participants. AUD was associated with increased platelet MAO-B activity [35]; therefore, this study included participants without AUD. As smoking significantly decreases platelet MAO-B activity ([31,38,39,41,52] present study), all participants were subdivided into smokers and non-smokers. The possible effect of various medication [31] was excluded because the study included participants who did not take MAO-B inhibitors and other medication affecting platelet MAO-B activity at the time or during the previous month.

Our study found that platelet MAO-B activity was significantly higher in veterans with PTSD compared with veterans without PTSD, in both smokers and non-smokers, but platelet MAO-B activity did not differ from the values in corresponding control subjects. This result is in contrast with the previous findings of lower platelet MAO-B activity in subjects with PTSD versus controls [36,53], or of similar platelet MAO-B activity in war veterans with or without PTSD [37]. In contrast to our data, decreased platelet MAO-B activity was found compared with control subjects [36,53]. The explanation for the divergent results lays in the fact that these studies included much smaller numbers of participants: only 23 patients with PTSD and 19 male controls [36], or 63 veterans with PTSD and 43 controls [53], as compared with 559 subjects with PTSD, 62 combat exposed veterans who did not develop PTSD, and 432 non-combat exposed healthy male controls included in this present study. In addition, when the authors controlled for the possible history of alcohol abuse, and further subdivided the PTSD group into those with a history of alcohol abuse (*N* = 14) and those without history of alcohol abuse (*N* = 9), significantly lower MAO activity was observed only in alcoholic PTSD group [36]. These findings might suggest that subjects with PTSD with lower platelet MAO-B activity [36] were mostly smokers with alcohol dependence. In contrast to these data, platelet MAO-B activity was similar in subjects with AUD subdivided into those with or without comorbid PTSD [51], while subjects with AUD without PTSD had significantly increased platelet MAO-B activity, controlled for the effects of smoking, liver diseases, and medication [35]. Our own previous study, which found similar platelet MAO-B activity between veterans with or without PTSD, also included a small number of participants: 31 soldiers with PTSD, 21 war veterans without PTSD, and 22 prisoners of war with PTSD [37]. These studies [36,37,53] did not control for the effect of smoking and liver disease on platelet MAO-B activity, which significantly reduce platelet MAO-B activity. Therefore, the small sample size, as well as the presence of liver disease and effect of smoking could explain these differences.

We have detected significantly higher platelet MAO-B activity in veterans (smokers and non-smokers) with more severe traumatic symptoms, evaluated using the CAPS scores, compared with veterans with mild or moderate traumatic symptoms. In confirmation, platelet MAO-B activity was significantly positively correlated with total scores in the CAPS subscales: re-experiencing, avoidance, and hyperarousal, in both smokers as well as non-smokers. Therefore, our data of the higher platelet MAO-B activity in subjects with PTSD might be explained in that, in contrast to previous studies, the present study included more veterans with severe PTSD (71%) compared with veterans with mild (7.7%) or moderate (21.6%) symptoms, and that more severe PTSD symptoms are related to increased platelet MAO-B activity. This result is in line with our previous data obtained on a smaller group of veterans with PTSD where there was a trend of higher platelet MAO-B activity associated with PTSD symptom severity according to CAPS score categories; however, this result was not significant [39]. Therefore, more severe traumatic symptoms (re-experiencing, avoidance, and hyperarousal symptoms) are related to increased platelet MAO-B activity. Our finding of increased platelet MAO-B activity in veterans with PTSD compared with veterans without PTSD, in both smokers and non-smokers, is in line with previous findings obtained in much smaller groups [38], which included 103 war veterans with PTSD, subdivided into those with (*N* = 25) or without (*N* = 78) psychotic features; 41 combat exposed veterans without PTSD; and 242 healthy control male subjects [38].

It is assumed that platelet MAO-B activity is reduced in different psychopathological behaviors and symptoms [21,30,31,32,33]. In line with this, our study detected decreased platelet MAO-B activity in veterans with PTSD with pronounced positive, psychotic, and depressive symptoms. It was proposed that, in adults, platelet MAO-B activity might indicate alterations in the central monoaminergic activity, while stress exposure affects the serotonergic system, leading to disturbances in the inhibitory regulation of aggressive, violent, and impulsive behaviors [54]. In contrast to our previous data, i.e., higher platelet MAO-B activity in smokers and non-smokers with psychotic PTSD compared with corresponding veterans without PTSD or veterans with non-psychotic PTSD [38], in the present study, both positive symptoms and psychotic symptoms were associated with decreased platelet MAO-B activity. The differences are in the diagnostic criteria for psychotic symptoms, as the previous study [38] determined psychotic symptoms as hallucinations or delusions on the psychotic module of the SCID, or specific disturbance in the form of thoughts by mental status examination, while our present study evaluated psychotic symptoms according to the defined cutoff in the PANSS-psychotic subscale including items P1, P2, P3, and P6. Although platelet MAO-B activity was increased in smokers with PTSD with severe psychotic symptoms, compared with smokers with PTSD with mild or moderate psychotic symptoms, that study included a smaller group (*N* = 249) of veterans with PTSD, and this result was not confirmed in non-smokers [39]. Different results might also be explained by the fact that our previous study [39] did not control for the influence of liver diseases on platelet MAO-B activity. To confirm this finding, we evaluated platelet MAO-B activity in all of the individual items listed in the PANSS-positive and PANSS-psychotic subscales, and found reduced platelet MAO-B activity in smokers and non-smokers with PTSD with severe delusions, conceptual disorganization, hallucinatory behavior, and excitement symptoms compared with corresponding groups with mild symptoms.

Reduced platelet MAO-B activity related to delusions and hallucinatory behavior in PTSD is in line with the older data that reported significantly lower platelet MAO-B activity in male patients with the paranoid subtype of schizophrenia, auditory hallucinations, and paranoia [55], or with the presence of hallucinations [56] or auditory hallucinations in schizophrenia [57] compared with controls. Discriminant function analyses revealed that platelet MAO-B activity was associated with excitement and disorientation according to the Brief Psychiatric Rating Scale (BPRS) in schizophrenia [56]. Low platelet MAO-B activity was detected in patients with paranoid schizophrenia characterized by the presence of auditory hallucinations and delusions versus controls [58]. A meta-analysis confirmed these results of the significantly lower platelet MAO-B activity associated with hallucinations and paranoid symptoms [59]. In addition, low platelet MAO-B activity was found in a large group of boys in the general population with high sensation seeking, impulsivity, and monotony avoidance [60], as well as in adult criminal offenders, which was not related to poor impulse control and increased aggressive and suicidal behavior, but usually associated with commitments of the violent acts [61]. Therefore, the presence of the more severe delusions, conceptual disorganization, hallucinatory behavior, and excitement symptoms was associated with decreased platelet MAO-B activity in our veterans with PTSD.

In our study, agitation, determined using the PANSS-excitement subscale, was not related to altered platelet MAO-B activity in smokers and non-smokers with PTSD. This result was unexpected because agitation was shown to be related to decreased platelet MAO-B activity in smokers with PTSD [39], but this effect was not confirmed in non-smokers. Platelet MAO-B was increased in agitated male, drug-naive patients with schizophrenia and adolescents with conduct disorder [52], but higher platelet MAO-B activity was detected only in severely agitated smokers compared with non-agitated smokers, and not in non-smokers. Compared with 27% severely agitated subjects [52] or 23% severely agitated veterans with PTSD [39] included in our previous studies, our study included less than 20% of severely agitated veterans with PTSD, and these results might suggest that severe excitement or agitation was not so frequent in this group of veterans with PTSD who were treatment-seeking and sampled in the hospital. In contrast to our results, higher platelet MAO-B activity was found in aggressive male youth living in juvenile detention, with or without conduct disorder; however, it was not associated with dissociative/aggressive/delinquent behavior, but only with the scores of verbal aggression, evaluated using the Overt Aggression Scale-Modified verbal aggression subscale [34]. On the other hand, lower platelet MAO-B activity was detected in incarcerated juvenile delinquents with high levels of novelty seeking, characterized by impulsivity, sensation seeking, and elevated exploratory activity [33], or in violent offenders from the prison [61]. Differences in the diagnoses (PTSD vs. schizophrenia and conduct disorder vs. delinquent/criminal behavior) and age of the subjects (adult vs. youth) might explain some of the divergent results. Although our veterans with PTSD did not have AUD, similarly to our results, platelet MAO-B activity did not differ between aggressive and non-aggressive subjects with AUD [35,62]. To confirm this unexpected result, we have evaluated platelet MAO-B activity in the individual items included in the PANSS-excitement subscale. Platelet MAO-B activity was not higher in any of the severe compared with mild symptoms included in the PANSS-excitement subscale (excitement, hostility, tension, uncooperativeness, poor impulse control) in our veterans with PTSD. We found that platelet MAO-B activity did not differ between smokers and non-smokers with severe and mild symptoms of hostility, and between non-smokers with severe or mild poor impulse control. This corresponds to no correlation between platelet MAO-B activity and the scores of impulse control, acts of violence, or suicide risk scales in subjects imprisoned because of violent crimes in male offenders or in male controls [61]. In our study, reduced platelet MAO-B activity was detected in smokers and non-smokers with PTSD with severe compared with mild excitement and tension symptoms. Although it is difficult to compare the results across studies because of different diagnoses and different methods of evaluation of symptoms, reduced platelet MAO activity was associated with risk activity such as bullfighting and thrill- and adventure-seeking in bullfighters compared with a control group [63]. Lower platelet MAO-B activity was found in smokers with severe versus mild poor impulse control in our PTSD veterans, while in drunken male drivers, i.e., subjects with socially deviant behavior, dysfunctional impulsivity was associated with low platelet MAO-B activity only in non-smokers and ex-smokers [64]. Regarding uncooperativeness of the PANSS-excitement subscale, in our study, veterans with PTSD had only severe uncooperativeness symptoms. In summary, this individual PANSS-excitement subscale item analysis revealed that severe agitation, consisting of pronounced symptoms of excitement, hostility, tension, uncooperativeness, and poor impulse control, was related to either decreased or similar platelet MAO-B activity in large groups of smokers and non-smokers with PTSD, resulting in no association between platelet MAO-B activity and agitation.

Our veterans with more severe depressive symptoms evaluated by the PANSS-depressive subscale (G1 = somatic concern; G2 = anxiety; G3 = guilty feelings; G6 = depression), compared with those with mild depressive symptoms, had lower platelet MAO-B activity. As the exclusion criterion of this study was the diagnosis of major depression, although depressive symptoms and PTSD are prevalent in trauma survivors [65], major depression was not present before the traumatic exposure and development of PTSD. Individual item analysis revealed that platelet MAO-B activity was significantly reduced in veterans, in both smokers and non-smokers, with more severe anxiety and depression symptoms compared with those with mild symptoms, while the severity of somatic concern and guilty feelings was not associated with altered platelet MAO-B activity. In contrast to our data, patients with generalized anxiety disorder or major depression had similar platelet MAO-B activity, and there was no correlation between platelet MAO-B and clinical severity of anxiety, determined using the Hamilton anxiety rating scale [66], and depression, panic disorder, or social phobia was not associated with platelet MAO-B activity in a large Australian sample, when controlled for the effect of smoking [50]. In the middle-aged non-clinical population, high platelet MAO-B was associated with higher anxiety and somatization [67]. The differences in findings might be explained in the different diagnostic groups.

In our study, we found increased platelet MAO-B activity in severe traumatic symptoms, and decreased platelet MAO-B activity in more pronounced positive, psychotic, and depressive symptoms in smokers and non-smokers with PTSD compared with values in the corresponding individuals with mild symptoms. Namely, MAO-B oxidizes dopamine and tyramine, but in platelets, MAO-B might also degrade serotonin and norepinephrine [20]. Platelets are used as a peripheral model for psychiatric [22,23,24,25,26,68] and neurologic [27] disorders, while platelet MAO-B activity was proposed to indicate disturbances in the central serotonergic functions [31,54]. Therefore, both increased and decreased MAO-B might be associated with monoaminergic alterations, leading to different psychopathological traits or disorders [69]. This could explain both higher and lower platelet MAO-B activity found in the present study and in individuals with different neuropsychiatric conditions and psychopathological behaviors compared with control subjects [33,34,35,52,70,71,72]. Therefore, platelet MAO-B is a promising biological marker related to psychopathology, because it degrades important neurotransmitters and because lower platelet MAO-B activity is frequently detected in different psychopathological conditions.

It should be mentioned that there is a link between glucocorticoid levels and MAO-B activity. In some mental disorders (major depressive disorder and anxious depression), which are frequently comorbid with PTSD, increased cortisol levels are associated with upregulated expression of MAO [73,74,75]. This association is achieved via interactions of different growth factors, glucocorticoid response element 4, and transcription factors that regulate glucocorticoid activation of MAO-B [76]. The interaction between the hormonal system, i.e., the hypothalamic–pituitary–adrenal axis, and monoaminergic systems was proposed to explain the relationship between altered MAO-B and cortisol levels [73]. This possible mechanism includes the following: increased tyrosine transaminase, decreased tyrosine and noradrenaline, and reduced activity of the noradrenergic system, on one hand, and increased tryptophan pyrrolase, decreased tryptophan and serotonin, and decreased activity of the serotonergic system, on the other hand. Reduced activities of the noradrenergic and serotonergic system induce elevations of cortisol levels. Increased cortisol levels increase MAO activity, and consequently elevate MAO-induced deamination products, i.e., hydrogen peroxide and free radicals. Lower levels of serotonin and noradrenaline are associated with increased MAO activity [73,74]. Therefore, deficiencies in monoamines are related to higher cortisol levels. Elevated cortisol is associated with activated MAO, and greater MAO activity is related to greater deamination of serotonin, noradrenaline, and dopamine, as well as to impaired biosynthesis of these monoamines. Hence, there is a close relationship between hormones and the monoaminergic system in the etiopathogenesis of mental disorders [73,74]. Additionally, the association between MAO-A and glucocorticoids was confirmed in an animal model [77]. Namely, the authors have postulated a hypothesis that, in PTSD, there is increased activity of the enzyme that metabolizes glucocorticoids, 11β-hydroxysteroid dehydrogenase-2, and of the main metabolizers of glucocorticoids (cortisol in humans and corticosterone in rats), a hepatic-microsomal oxidation enzyme, CYP3A; on the other hand, there is decreased activity of the enzyme that degrades glucocorticoids, 11β-hydroxysteroid dehydrogenase-1 [77]. These alterations lead to increased 11-dehydrocorticosterone levels and decreased basal glucocorticoid levels. Reduced cortisol levels are associated with suppression of MAO-A activity and expression in the prefrontal cortex and other brain regions, with subsequent enhanced glucocorticoid metabolism, and consequent accumulation of noradrenaline, resulting in the deficient inhibitory activity of the prefrontal cortex through hyperactivation of amygdala [77]. Therefore, decreased glucocorticoid levels are associated with increased oxidative stress, inflammation, adrenal insufficiency, altered metabolism of glucocorticoid metabolizing enzymes, decreased brain MAO-A activity, increased noradrenaline, downregulation of prefrontal cortex, amygdala hyperactivation, increased density of the tissue glucocorticoid receptors, upregulation of the corticotrophin releasing factor (CRH), blunted ACTH response to CRH stimulation, and blunted adrenal response to ACTH stimulation [77]. All of these alterations are suggested to be associated with progression of PTSD [77]. In our study, we found higher platelet MAO-B activity in veterans with PTSD with severe compared with mild traumatic symptoms, and on the other hand, lower platelet MAO-B activity in veterans with severe versus mild positive, psychotic, and depressive symptoms. Unfortunately, we did not determine cortisol levels in these participants to confirm the above-mentioned hypotheses. Subjects with PTSD have reduced daily cortisol output; increased noradrenaline, CRH, and proinflammatory cytokine concentrations; reduced glucocorticoid signaling; and elevated glucocorticoid responses and sensitivity [5]; therefore, a disrupted HPA axis might be associated with altered MAO-B activity in PTSD.

The limitations of the study should be acknowledged. As only the male population of veterans with combat-related PTSD was included, inclusion of female subjects and civilian population with PTSD is advised in further studies.

The strength of the study is a large number of subjects (*N* = 1053); the fact that platelet MAO-B activity was determined in one center, excluding possible methodological issues [50]; and that it was controlled for the effect of sex, smoking, liver diseases, ethnicity, medication, and presence of different symptoms.

## 5. Conclusions

This study detected reduced platelet MAO-B activity in both smokers and non-smokers with PTSD with severe positive, psychotic, and depressive symptoms, and this finding was confirmed as platelet MAO-B was mostly lower in all subjects with severe compared with mild individual positive, psychotic, and depressive symptoms. Reduced MAO-B activity can lead to higher dopamine availability in the prefrontal cortex, which might be associated with more pronounced positive, psychotic, and depressive symptoms in PTSD. On the other hand, platelet MAO-B activity was significantly increased in smokers and non-smokers with PTSD with severe traumatic symptoms compared with groups with mild and moderate symptoms. There are bidirectional interactions between trauma and dopaminergic alterations that affect regions affected by PTSD, primarily the medial prefrontal cortex and its regulation of the limbic system and modulation of emotions [18]. Low platelet MAO-B was reported to represent a nonspecific marker indicating predisposition to general psychopathology [33]. Therefore, altered platelet MAO-B activity was confirmed to be associated with the development of and severity of different symptoms occurring in PTSD.

## Figures and Tables

**Table 1:** CAPS scores in the group of war veterans with PTSD.

CAPS Item	Scores
Criterion B: re-experiencing	29 (22; 30)
Criterion C: avoidance	44 (34; 45)
Criterion D: hyperarousal	31 (26; 33)
Total CAPS scores	105 (79; 107)

CAPS = Clinician-Administered PTSD Scale; scores are expressed as median (Q1; Q3).

**Table 2:** PANSS scores of war veterans with PTSD according to different PANSS subscales.

PANSS Subscale	PANSS Items	Cutoff	Score	Number of Subjects with Severe Symptoms (%)
PANSS-positive	P1 + P2 + P3 + P4 + P5 + P6 + P7	28	12 (8; 14)	29 (5.2%)
PANSS-excitement	P4 + P7 + G4 + G8 + G14	14	12 (10; 13)	110 (19.7%)
PANSS-psychotic	P1 + P2 + P3 + P6	16	6 (4; 8)	44 (7.9%)
PANSS-depressed	G1 + G2 + G3 + G6	16	11 (9; 12)	51 (9.1%)

PANSS = the Positive and Negative Syndrome Scale; PANSS scores are expressed as median (Q1; Q3). Severe symptoms = PANSS score ≥ cut off; P1 = delusions; P2 = conceptual disorganization; P3 = hallucinatory behavior; P4 = excitement; P5 = grandiosity; P6 = suspiciousness/persecution; P7 = hostility; G1 = somatic concern; G2 = anxiety; G3 = guilty feelings; G4 = tension; G6 = depression; G8 = uncooperativeness; G14 = poor impulse control.

**Table 3:** Platelet MAO-B activity in smokers with PTSD subdivided into those with severe or mild symptoms evaluated by specific PANSS items.

PANSS Item	Symptoms	N	Platelet MAO-B Activity	Mann–Whitney U Test
P1	severe	33	15 (10.9; 22.3)	U = 8101.50 *p* < 0.001 *
	mild	335	28.8 (16.6; 45.1)	
P2	severe	33	15.0 (10.3; 22.3)	U = 8184.00 *p* < 0.001 *
	mild	335	28.8 (16.6; 45.1)	
P3	severe	38	15.3 (10.3; 22.37)	U = 9194.00 *p* < 0.001 *
	mild	330	28.8 (16.6; 45.1)	
P4	severe	43	15.7 (10.9; 24.3)	U = 9965.50 *p* < 0.001 *
	mild	325	29.1 (16.6; 45.1)	
P5	severe	3	12.7 (8.3; 28.1)	U = 815.50 *p* = 0.144
	mild	365	28.0 (15.7; 43.1)	
P6	severe	41	21.3 (12.7; 33.0)	U = 7819.50 *p* = 0.082
	mild	327	28.1 (16.2; 44.6)	
P7	severe	65	28.1 (15.0; 54.3)	U = 9302.50 *p* = 0.484
	mild	303	27.9 (16.0; 41.2)	
G1	severe	87	25.2 (13.7; 42.9)	U = 12,786.50 *p* = 0.516
	mild	281	28.0 (16.2; 43.1)	
G2	severe	125	18.3 (11.3; 33.4)	U = 20,206.00 *p* < 0.001 *
	mild	243	31.5 (19.6; 46.7)	
G3	severe	98	30.0 (16.9; 49.3)	U = 11,854.00 *p* = 0.127
	mild	270	25.5 (15.3; 41.1)	
G4	severe	160	21.5 (13.1; 38.9)	U = 19,994.50 *p* = 0.001 *
	mild	208	30.5 (18.5; 45.5)	
G6	severe	89	14.0 (9.4; 20.5)	U = 20,440.00 *p* < 0.001 *
	mild	279	33.5 (21.6; 49.7)	
G8	severe	0	/	/
	mild	368	27.9 (15.6; 43.0)	
G14	severe	57	21.7 (13.1; 35.2)	U = 10,723.00 *p* = 0.012 *
	mild	311	28.5 (16.2; 45.1)	

Platelet MAO-B activity is expressed as median (Q1; Q3). * Significant difference; P1 = delusions; P2 = conceptual disorganization; P3 = hallucinatory behavior; P4 = excitement; P5 = grandiosity; P6 = suspiciousness/persecution; P7 = hostility; G1 = somatic concern; G2 = anxiety; G3 = guilty feelings; G4 = tension; G6 = depression; G8 = uncooperativeness; G14 = poor impulse control.

**Table 4:** Platelet MAO-B activity in non-smokers with PTSD subdivided into those with severe or mild PANSS individual items.

PANSS Item	Symptoms	N	Platelet MAO-B Activity	Mann–Whitney U Test
P1	severe	15	19.1 (12.4; 32.3)	U = 1744.00 *p* = 0.039 *
	mild	176	31.0 (18.5; 51.0)	
P2	severe	14	16.5 (12.4; 31.7)	U = 1695.00 *p* = 0.022 *
	mild	177	31.1 (18.8; 50.6)	
P3	severe	18	15.6 (12.1; 27.5)	U = 2290.00 *p* = 0.001 *
	mild	173	31.8 (20.7; 51.3)	
P4	severe	17	15.7 (12.1; 25.8)	U = 2137.00 *p* = 0.002 *
	mild	174	31.5 (20.6; 50.6)	
P5	severe	2	29.5 (8.7; 50.3)	U = 226.00 *p* = 0.634
	mild	189	30.9 (17.8; 48.7)	
P6	severe	26	27.0 (15.7; 40.1)	U = 2413.00 *p* = 0.306
	mild	165	31.1 (18.0; 50.6)	
P7	severe	25	30.2 (15.4; 60.8)	U = 2115.00 *p* = 0.877
	mild	166	31.0 (18.4; 46.6)	
G1	severe	26	27.7 (12.4; 61.5)	U = 2281.00 *p* = 0.604
	mild	165	30.9 (18.8; 46.6)	
G2	severe	63	18.7 (11.7; 31.9)	U = 5952.50 *p* < 0.001 *
	mild	128	35.2 (24.9; 56.8)	
G3	severe	40	35.1 (14.3; 52.0)	U = 2914.00 *p* = 0.733
	mild	151	30.3 (18.4; 46.6)	
G4	severe	76	22.1 (12.3; 36.6)	U = 5921.00 *p* < 0.001 *
	mild	115	34.6 (24.8; 53.0)	
G6	severe	42	16.7 (11.6; 25.0)	U = 4923.00 *p* < 0.001 *
	mild	149	33.7 (24.0; 55.5)	
G8	severe	0	/	/
	mild	191	30.9 (17.6; 48.9)	
G14	severe	27	30.9 (17.2; 60.3)	U = 2171.00 *p* = 0.872
	mild	164	30.9 (17.7; 48.8)	

Platelet MAO-B activity is expressed as median (Q1; Q3). * Significant result; P1 = delusions; P2 = conceptual disorganisation; P3 = hallucinatory behaviour; P4 = excitement; P5 = grandiosity; P6 = suspiciousness/persecution; P7 = hostility; G1 = somatic concern; G2 = anxiety; G3 = guilty feelings; G4 = tension; G6 = depression; G8 = uncooperativeness; G14 = poor impulse control.

## Data Availability

Not applicable.
